# Clinical implications for Vascular Endothelial Growth Factor in the lung: friend or foe?

**DOI:** 10.1186/1465-9921-7-128

**Published:** 2006-10-17

**Authors:** Andriana I Papaioannou, Konstantinos Kostikas, Panagoula Kollia, Konstantinos I Gourgoulianis

**Affiliations:** 1 Respiratory Medicine Department, University of Thessaly School of Medicine, University Hospital of Larissa, Larissa 41110, Greece; 2 Biology Department, University of Thessaly School of Medicine, University Hospital of Larissa, Larissa 41110, Greece

## Abstract

Vascular endothelial growth factor (VEGF) is a potent mediator of angiogenesis which has multiple effects in lung development and physiology. VEGF is expressed in several parts of the lung and the pleura while it has been shown that changes in its expression play a significant role in the pathophysiology of some of the most common respiratory disorders, such as acute lung injury, asthma, chronic obstructive pulmonary disease, obstructive sleep apnea, idiopathic pulmonary fibrosis, pulmonary hypertension, pleural disease, and lung cancer. However, the exact role of VEGF in the lung is not clear yet, as there is contradictory evidence that suggests either a protective or a harmful role. VEGF seems to interfere in a different manner, depending on its amount, the location, and the underlying pathologic process in lung tissue. The lack of VEGF in some disease entities may provide implications for its substitution, whereas its overexpression in other lung disorders has led to interventions for the attenuation of its action. Many efforts have been made in order to regulate the expression of VEGF and anti-VEGF antibodies are already in use for the management of lung cancer. Further research is still needed for the complete understanding of the exact role of VEGF in health and disease, in order to take advantage of its benefits and avoid its adverse effects. The scope of the present review is to summarize from a clinical point of view the changes in VEGF expression in several disorders of the respiratory system and focus on its diagnostic and therapeutic implications.

## Background

Over the past few years extensive research has been done on the role of vascular endothelial growth factor (VEGF) in several physiologic and pathologic conditions in the lung. VEGF is a pluripotent growth factor that is critical for lung development and has multiple physiological roles in the lung, including the regulation of vascular permeability and the stimulation of angiogenesis. Increasing evidence in the current medical literature suggests that VEGF additionally plays significant role in the development of several lung disorders, including lung cancer, chronic obstructive pulmonary disease (COPD), pulmonary hypertension (PH) and acute lung injury (ALI) [1]. However, in many of these disorders the role of VEGF is not clear, as contradictory reports suggest both protective and deleterious mechanisms of action. The aim of the present review is to summarize the changes on the expression of VEGF in the lung and the pleura in several pathologic conditions of the respiratory system, and to focus on the diagnostic and therapeutic implications of VEGF in lung diseases.

### What is VEGF?

VEGF is one of the most potent mediators of vascular regulation in angiogenesis and vascular permeability to water and proteins [2]. VEGF is believed to increase vascular permeability 50,000 times more than does histamine [3]. It has been also reported that VEGF induces fenestration in endothelial cells both in vivo and in vitro [4]. Over the past few years several members of the VEGF gene family have been identified, including VEGF-A, VEGF-B, VEGF-C, VEGF-D, VEGF-E, and placental growth factor (PLGF) [5]. The most studied molecule of the VEGF family is VEGF-A, also referred as VEGF.

The human VEGF gene is localized in chromosome 6p21.3 [6] and is organized in eight exons, separated by seven introns [5]. Human VEGF isoforms include 121, 145, 165, 183, 189 and 206 amino acids (VEGF_121_, VEGF_145_, VEGF_165_, VEGF_183_, VEGF_189_, and VEGF_206_, respectively), which all come from alternative exon splicing of one single VEGF gene [5]. Due to its bioactivity and biological potency, VEGF_165 _is the predominant isoform of VEGF [7]. Native VEGF is a basic, heparin binding, homodimeric glycoprotein of 45 kDa [6].

The biological activity of VEGF is dependent on its reaction with specific receptors. Three different receptors have been identified that belong to the tyrosine-kinase receptor family: VEGFR-1/Flt-1, VEGFR-2/Flk-1 (KDR), and VEGFR-3 (Flt-4). Both VEGFR-1 and VEGFR-2 have extracellular immunoglobulin-like domains as well as a single tyrosine kinase transmembrane domain and are expressed in a variety of cells [7]. VEGFR-3 is a member of the same family but it is not a receptor for VEGF as it binds only VEGF-C and VEGF-D [5]. VEGFR-3 is predominantly expressed in the endothelium of lymphatic vessels. Neuropilin-1, a receptor for semaphorins in the nervous system, is also a receptor for the heparin-binding isoforms of VEGF and PIGF. However, there is no evidence that neuropilin signals after VEGF binding. It has been proposed that neurophilin-1 presents VEGF_165 _to Flk-1/KDR in a manner that enhances the effectiveness of Flk-1/KDR signal transduction [6].

### Transcriptional and post transcriptional regulation of VEGF

VEGF gene expression is known to be regulated by several factors, including hypoxia, growth factors, cytokines and other extracellular molecules [8]. Hypoxia plays a key role in VEGF gene expression both in vivo and in vitro, while VEGF mRNA expression is induced after exposure to low oxygen tension [6]. Hypoxia-induced transcription of VEGF mRNA is apparently mediated by the binding of hypoxia-inducible factor 1 (HIF-1) to an HIF-1 binding site located in the VEGF promoter [8]. In addition to the induction of VEGF gene transcription, hypoxia also promotes the stabilization of VEGF mRNA, which is labile under conditions of normal oxygen tension, by proteins that bind to sequences located in the 3' untranslated region of the VEGF mRNA [9,10]. There is also evidence that the hypoxia-mediated elevation in VEGF transcription is also mediated by sites that are found in the 5' untranslated region of the VEGF mRNA [8]. Except the HIF-1 transcription binding side, VEGF promoter region has several potential transcription factor binding sites such as AP-1, AP-2, Egr-1, Sp-1 and many others which are also involved in VEGF transcription regulation [11].

The human VEGF gene contains two hypoxia-sensitive enhancer elements and several consensus binding sites for growth factor regulated transcription factors [12]. The presence of these regulatory sequences suggest the synergistic effect of boyh hypoxia and growth factors at the level of transcription [12]. Growth factors that can stimulate VEGF production include epidermal growth factor (EGF), transforming growth factor β (TGF-β), keratinocyte growth factor (KGF) and insulin like growth factor (IGF) [5,8]. These observations suggest that the paracrine or autocrine release of such factors cooperates with local hypoxia in regulating VEGF release in the microenvironment [5].

The major cytokines that induce VEGF expression are interleukin 1α (IL-1α), and interleukin 6 (IL-6) [5]. However, other cytokines such as interleukin 10 (IL-10) and interleukin 13 (IL-13) down-regulate VEGF expression [8]. Finally, it has been shown that prostaglandin E_2_, thyroid stimulating hormone (TSH), and adrenocorticotropic hormone (ACTH) can also increase the expression of VEGF mRNA [6].

Other studies have shown that the product of the von Hippel-Lindau (VHL) tumor suppressor gene plays an important role in HIF-1 dependent hypoxic responses and provides negative regulation of many hypoxia inducible genes, including VEGF gene [5]. VHL inhibition of VEGF expression is mediated by transcriptional and post transcriptional mechanisms. At the transcriptional level, VHL forms a complex with the Sp1 transcription factor and inhibits Sp1-mediated VEGF expression as a result of the binding of Sp1 to a specific region in the VEGF promoter [8]. At the post transcriptional level, VHL inhibits the activity of several protein kinases which stabilize VEGF mRNA. It is known that mutations in the VHL gene are associated with VEGF overexpression and increased angiogenesis [8].

### Interdependence of VEGF with other angiogenic factors

Vascular development is the result of collaboration between three different families of growth factors: VEGFs, angiopoietins and ephrins [13]. Incorporation of those three different kinds of growth factors in a model of vascular formation has showed that VEGF initiates the formation of vascular vessels by vasculogenesis or angiogenic sprouting both during development and in the adult. Angiopoietin-1 and ephrin B_2 _are required for further remodeling and maturation of this initially immature vasculature [14]. It has been reported that VEGF administration in animal models promotes by itself only leaky, immature and unstable vessels. Administration of angiopoietin-1 stabilizes and protects the adult vasculature making it resistant to the damage and leak induced by VEGF or inflammation [14]. Existing data suggest that VEGF and angiopoietins act in a very complementary and coordinated fashion [13]. Finally, the ephrins, are acting in later stages of vascular development though they may also contribute somewhat to the formation of vessel primordia [13]. It is important that all of these factors must collaborate in perfect harmony to form functional vessels [14].

### The role of VEGF in lung development

The formation of lung's vasculature includes three processes: angiogenesis, which gives rise to the central vessels via the sprouting of new vessels from preexisting ones; vasculogenesis, which provides the peripheral vessels via the formation of capillaries from blood lakes; and fusion between the central and peripheral systems to create the pulmonary circulation. A likely candidate as a regulator for the formation of the lung's vasculature in all three phases is VEGF [15]. High levels of VEGF protein and mRNA have been detected in the developing lung, suggesting that VEGF plays a central role in the formation of lung vasculature and also in the epithelial-endothelial interactions that are critical for normal lung development [16].

The expression of VEGF mRNA and protein is localized to the distal airway epithelial cells in the midtrimester human fetal lung and their levels increase with time; [16] in contrast, VEGF levels are decreased in human infants with bronchopulmonary dysplasia (BPD). Furthermore, the inhibition of the VEGF receptors in the immature lung reduces eNOS expression and NO bioactivity and later leads to the development of the structural and functional features of BPD [17]. Finally, VEGF stimulates surfactant production by alveolar type II cells, which results in lung maturation and protects from the development of respiratory distress syndrome of the newborn [18].

### VEGF protein and VEGF receptors in the lung and the pleura

Although VEGF has been characterized as a mitogen for vascular endothelial cells, recent studies identified the presence of VEGF and its receptors in several cell types in many organs. It has been reported that lung presents the highest level of VEGF gene expression among normal tissues [19]. VEGF and its receptors (VEGFR-1, VEGFR-2 and NRP1) have been detected in alveolar type II cells, airway epithelial cells, mesenchymal cells, airway and vascular smooth muscle cells, macrophages and neutrophils [7,20]. In healthy human subjects, VEGF protein is composed in the lung and VEGF protein levels in alveoli are 500 times higher than in plasma [21]. It has been proposed that the high levels of VEGF protein on the respiratory epithelial surface may function as a physiological reservoir [21]. Potential cellular sources of VEGF include alveolar and airway epithelial cells [22], as well as airway smooth muscle cells [21]. Normal lung alveolar macrophages produce very small amounts of VEGF. Additionally, although neutrophils carry intracellular pools of VEGF, their number in normal lung is very low [7]. Therefore, neither of those two types of cells is likely to affect VEGF levels in alveoli in health. In normal lung, VEGF may slowly diffuse across the alveolar epithelium to the adjacent vascular endothelium and act in a paracrine fashion [7]. However, in disease states, the expression of VEGF or its receptors is affected, and that is often related to the pathophysiology and the particular characteristics of each disease. In addition, human mesothelial cells are known to be a source of elevated concentrations of VEGF in the pleural fluid [23], and these cells have also been shown to be positive for VEGFR-1 [24].

### Where can VEGF be measured?

VEGF has been measured in several kinds of biological fluids and cells of the lung parenchyma. The most common origins used for its measurements are blood (serum or plasma), bronchoalveolar lavage (BAL) fluid, sputum, bronchial epithelial cells, alveolar type II cells, alveolar macrophages, neutrophils, endothelial cells of the alveolar capillaries, and airway or vascular smooth muscle cells.

It is important to point out that serum VEGF levels are higher compared to those measured in plasma. The reason for that difference, is that serum VEGF reflects *ex vivo *platelet and leukocyte release during blood clotting, thus resulting in an increase of VEGF concentrations by 2- to 7-fold [25]. In BAL fluid, VEGF levels actually correspond to VEGF levels of the epithelial lining fluid. To estimate VEGF concentrations in epithelial lining fluid, investigators have taken into account the generally accepted estimate that pooled BAL fluid is diluted 100 times compared with alveolar fluid [22]. In healthy human subjects, epithelial lining fluid VEGF protein levels are 500 times higher than plasma levels [21].

## VEGF in diseases of the lung and the pleura

The consequences of the administration or inhibition of VEGF have been widely studied in animal models (Table 1). In humans, elevated or reduced VEGF levels have been found in various respiratory disorders (Table 2) and have been associated with various clinical manifestations of those disease entities (Table 3). A detailed description of the role of VEGF in diseases of the lung and the pleura follows.

**Table 1:** Effects of VEGF administration or inhibition in animal models.

**Intervention**	**Result**	**Reference**
Provocation of intratracheal VEGF overexpression in mice	Dose-dependent increase in extravascular lung water intra-alveolar edema, and increased pulmonary capillary permeability.	[19]
Administration of a VEGFR inhibitor in mice	Decrease in bronchial hyperresponsiveness and migration of inflammatory cells through the endothelial basement membrane and reduction of VEGF-induced plasma leakage.	[42]
Intraperitoneal administration of a VEGF receptor blocker in rats	Induction of alveolar septal cell apoptosis and enlargement of air spaces (emphysema).	[46]
VEGF gene transfer in immature rabbits	Reduction of bleomycin-induced pulmonary hypertension.	[78]
Blockade of VEGF activity in malignant pleural effusion model in mice	Decrease of vascular permeability and reduction of pleural fluid.	[3, 113]

VEGF: vascular endothelial growth factor; VEGFR: vascular endothelial growth factor receptor.

**Table 2:** VEGF levels in various respiratory disorders.

**Disease**	**VEGF levels**	**Reference**
ALI/ARDS	Elevated plasma VEGF levels. Reduced VEGF levels in the epithelial lining fluid.	[27] [28]
Asthma	Increased VEGF levels in induced sputum. Increased VEGF levels in BAL fluid. Increased VEGF-positive cells in bronchial biopsies.	[4, 32, 33] [34] [35, 36]
COPD	Increased VEGF expression in bronchial, bronchiolar and alveolar epithelium; bronchiolar macrophages; airway and vascular smooth muscle cells of bronchiolar and alveolar regions.	[43, 48]
	Increased VEGF concentrations in induced sputum in chronic bronchitis.	[45]
	Reduced VEGF concentrations in induced sputum in emphysema.	[45]
Obstructive sleep apnea	Increased serum and plasma VEGF levels.	[25, 53-55]
Idiopathic Pulmonary Fibrosis	Plasma VEGF concentrations did not differ between patients with IPF and controls. Depressed BAL fluid VEGF concentrations.	[53] [60-62]
Tuberculosis	Increased circulating VEGF levels in patients with active pulmonary tuberculosis compared to healthy controls and patients with old tuberculosis.	[67, 68]
Pleural fluid	Higher VEGF levels in pleural effusions associated with malignancies compared to benign effusions. Higher VEGF levels in empyemas compared to uncomplicated parapneumonic effusions.	[24, 81, 82, 84, 85] [24, 89]
	Higher VEGF levels in tuberculous pleural effusions compared to transudates.	[90]
Lung cancer	Increased serum VEGF levels.	[98, 99]

VEGF: vascular endothelial growth factor; ALI/ARDS: acute lung injury/acute respiratory distress syndrome; COPD: chronic obstructive pulmonary disease; IPF: idiopathic pulmonary fibrosis; BAL: bronchoalveolar lavage.

**Table 3:** Associations of VEGF levels with clinical manifestations

**Disease**	**Associations of VEGF levels with clinical manifestations**	**Reference**
ALI/ARDS	Association of the time-course of plasma VEGF levels with patients' outcome; higher VEGF plasma levels were found in non-survivors. Association of increased epithelial lining fluid VEGF levels with recovery.	[27] [28]
Asthma	Negative correlation of increased VEGF levels in asthmatic patients with the degree of airway obstruction.	[4, 36]
COPD	Negative correlation between VEGF concentrations in sputum samples with airflow limitation (as expressed by FEV_1_) in patients with chronic bronchitis. Positive correlation of sputum VEGF levels with FEV_1 _and gas exchange (as measured by the DL_CO_) in patients with emphysema.	[45]
Obstructive sleep apnea	Correlation of circulating VEGF levels with the severity of OSA (as expressed by the apnea-hypopnea index) and with the degree of nocturnal desaturations.	[25, 53] [55]
Idiopathic Pulmonary Fibrosis	Correlation of plasma VEGF levels of with the extent of parenchymal involvement in HRCT. Correlation of VEGF concentrations in BAL fluid with DL_CO_.	[59] [60-62]
Tuberculosis	Higher serum VEGF levels in TB patients without cavitary lesions compared to those with typical chest cavities.	[70]
Lung Cancer	Correlation of the expression of VEGF with tumor size.	[98]
	Patients with higher serum VEGF levels had lower survival compared to patients with lower VEGF levels.	[96, 100-103]

VEGF: vascular endothelial growth factor; ALI/ARDS: acute lung injury/acute respiratory distress syndrome; COPD: chronic obstructive pulmonary disease; BAL: bronchoalveolar lavage; TB: tuberculosis; OSA: obstructive sleep apnea; HRCT: high resolution computed tomography.

### Acute lung injury and acute respiratory distress syndrome

The acute respiratory distress syndrome (ARDS) is the most extreme manifestation of acute lung injury (ALI) [26]. Pulmonary injury in ARDS results in the disruption of the alveolar-capillary membrane which leads to a severe dysfunction of gas exchange and chest radiographic abnormalities, following a predisposing injury and in the absence of heart failure [7]. The hallmarks of ALI are increased capillary permeability, interstitial and alveolar edema, influx of circulating inflammatory cells, and formation of hyaline membranes [7]. It is commonly believed that inflammatory mediators create an acute inflammatory response in the microvessels of the lung and that locally released inflammatory cell products damage the endothelial cells resulting in increased permeability [27]. A wide range of vasoactive agents is released and modulates vascular tone at a local level. The result is a loss of functional and structural vascular integrity. VEGF has been shown to play a key role in this process.

The potential role of VEGF in ARDS has been studied in both sides of the alveolar capillary interface [27,28]. It has been shown that plasma VEGF levels in subjects with ARDS were elevated compared to controls [27]. Additionally, the time-course of VEGF was associated to the patients' outcome, with VEGF plasma levels being higher in non-survivors compared to survivors [27]. Interestingly, increases in plasma VEGF over 100% baseline values were associated with 100% mortality [27]. The same authors consequently reported that VEGF levels in the epithelial lining fluid of patients with ARDS were significantly lower than in controls [28]. In contrast to plasma measurements, increasing epithelial lining fluid VEGF levels were associated with recovery [28]. The authors suggested that lung might represent a physiological reservoir of VEGF with potentially devastating effects if the epithelial barrier is breached [28].

Additionally, the intratracheal administration of VEGF has been shown to provoke a dose-dependent increase in extravascular lung water, while lung histology showed widespread intra-alveolar edema, and increased pulmonary capillary permeability [19]. According to the above, one could conclude that in the case of hydrostatic pulmonary edema, in which the alveolar capillary membrane is normal, VEGF levels in the pulmonary edema fluid should be higher than in the case of ALI/ARDS [29]. However, VEGF levels did not differ between patients with hydrostatic pulmonary edema and ALI/ARDS neither in the pulmonary edema fluid nor in plasma [29]. Those data suggest that a possible explanation for the decreased levels of alveolar VEGF in both ALI/ARDS and hydrostatic pulmonary edema may be the dilution caused from the alveolar flooding rather than the degree of lung injury [29].

In the early stage of lung injury different insults and proinflamatory cytokines stimulate the production and release of VEGF from type II cells, alveolar macrophages and neutrophils. Therefore, the epithelial-endothelial barrier is exposed to high concentrations of VEGF, which increases vascular permeability and leads to interstitial edema [7]. During the development of lung injury, damage of alveolar epithelial cells reduces the production of VEGF and leads to the low concentration detected in the BAL fluid of these patients. The release of VEGF from other organs and circulating leucocytes may additionally contribute to the increased serum concentration of VEGF in patients with ALI/ARDS [7]. Finally, during the recovery of lung injury, alveolar cells are being repaired and increased local production of VEGF may play a role in the repair and angiogenesis by acting on VEGFR-2 [7]. On the other hand it has been shown that VEGF production stimulated by IL-13 in transgenic mice leads to a protection against hyperoxic acute lung injury [30]. It has also been suggested that VEGF is critical for pulmonary angiogenesis, as it stimulates endothelial cell growth. It also seems to play a role in lung epithelial cell proliferation. According to that, regulation of VEGF synthesis in the lung may affect lung injury repair [22].

These observations indicate that the expression and function of VEGF in ALI/ARDS vary. The results of its biological activity depend on the pathophysiological conditions, the timing and the degree of epithelial and endothelial damage [7]. It is not clear whether VEGF acts as a cause for the development of ALI/ARDS or as a mediator that promotes recovery. However, no conclusion on the biological functions of VEGF in ALI/ARDS can be based only on measurements of its levels and further research is needed for the clarification of its role.

### Asthma

Bronchial asthma is physiologically characterized by variable airflow obstruction and airway hyperresponsiveness [31]. Some of the most common changes in asthmatic airway walls are epithelial desquamation, goblet cell hyperplasia, smooth muscle hypertrophy-hyperplasia, as well as growth and proliferation of new vessels [32]. Increased VEGF levels in induced sputum,[4,32,33] BAL fluid, [34] and VEGF-positive cells in bronchial biopsies [35,36] have been found in patients with asthma compared to healthy controls. The increased vascularity of bronchial mucosa in asthmatic subjects has been related to increased numbers of VEGF-positive cells, suggesting a pathogenic role for VEGF in the pathology of the asthmatic airway [36]. Additionally, the increased VEGF levels in asthmatic patients are negatively correlated with the degree of airway obstruction, and positively correlated with the degree of eosinophilic inflammation and an index indicative of vascular permeability [4,36]. This VEGF-related increased vascular permeability in the asthmatic airways has also been proposed as a mechanism that may be in part responsible for the exercise-induced bronchoconstriction in asthmatics [33]. In addition to its role in vascular permeability in the asthmatic mucosa, VEGF has been related to increased basement membrane thickness in biopsies from asthmatic patients, suggesting a possible role of VEGF in airway remodelling [37].

Treatment of asthmatic subjects with inhaled corticosteroids resulted in the decrease of VEGF levels in induced sputum; however, asthmatic patients after treatment had still higher VEGF levels in induced sputum than controls [4,32]. Inhibition of VEGF expression by corticosteroids has additionally been shown *in vitro *in airway smooth muscle and epithelial cell cultures [38,39]. Cysteinyl leukotriene receptor antagonists reduce VEGF expression in animal models of allergic asthma [40]. A decrease in induced sputum VEGF levels was also observed after treatment of steroid-naive asthmatics with pranlucast, a selective leukotriene receptor antagonist. However, the addition of pranlucast to inhaled corticosteroids added little efficacy to the reduction of airway VEGF levels [41]. In animal models it has also been shown that treatment with a VEGFR inhibitor resulted in reduction of VEGF-induced plasma leakage, decreased bronchial hyperresponsiveness and migration of inflammatory cells through the endothelial basement membrane [42].

### Chronic Obstructive Pulmonary Disease

Chronic obstructive pulmonary disease (COPD) is a disease state characterized by airflow limitation that is not fully reversible, usually progressive, and associated with an abnormal inflammatory response of the lungs in response to noxious particles and gases [43]. However COPD does not seem to be a single entity. Its two major subtypes are chronic bronchitis and emphysema and lead in the two clearly distinguishable phenotypes of the "blue bloater" and the "pink puffer" [44]. The role of VEGF in the development of the different phenotypes of COPD has been widely investigated. In vitro studies have shown that cigarette smoke decreases the expression and signaling of VEGF and VEGF receptors and may result in emphysema due to pulmonary endothelial death, followed by progressive disappearance of the alveolar septum due to apoptosis [45]. It has also been shown that inhibition of VEGF receptors induced alveolar septal cell apoptosis and led to enlargement of the air spaces, indicative of emphysema [46]. Inhibition of VEGF receptors additionally resulted in an increase of markers of oxidative stress which plays central role in the development of COPD [47].

Studies have shown that COPD is associated with increased expression of VEGF in the bronchial, bronchiolar and alveolar epithelium and in bronchiolar macrophages, as well as in airway and vascular smooth muscle cells in both the bronchiolar and alveolar regions [43,48]. VEGF receptors were also increased in patients with COPD compared with non-COPD subjects [43]. Histological examinations of lungs with emphysema have shown that the alveolar walls in centrilobular emphysema appear to be remarkably thin and almost avascular [46]. On the contrary, in case of chronic bronchitis bronchial vascularity is increased [45]. Those differences in the vascularity of the airways in the two different manifestations of COPD are reflected in the VEGF levels in induced sputum of COPD patients. VEGF concentrations were found significantly elevated in patients with chronic bronchitis compared to controls, whereas they were significantly reduced in patients with emphysema [45]. In a similar pattern with asthmatic subjects, patients with chronic bronchitis presented a negative correlation between the concentrations of VEGF in sputum samples and airflow limitation, as expressed by FEV_1_. In contrast, there was a positive correlation of sputum VEGF levels with FEV_1 _and gas exchange (as measured by the DL_CO_) in patients with emphysema [45]. Another study suggested an inverse role between VEGF and oxidative stress in COPD, as VEGF levels were reduced and a reciprocal increase in oxidative stress was observed with the increased severity of the disease [49]. A possible mechanism connecting the two findings might be that epithelial cell injury mediated by oxidative stress may induce the decrease in lung VEGF levels, resulting in promotion of the development of COPD. The above studies provided a link between VEGF levels and the development of chronic bronchitis and emphysema; yet, it remains to be clarified whether VEGF represents a cause or a consequence in these mechanisms.

The significance of VEGF expression in patients with COPD remains controversial. Increased bronchial vasculature could induce inflammatory cell trafficking and exudation and transudation of mediators, particularly if vascular permeability was altered; additionally, it could also contribute to airway hyperresponsiveness by supporting the increased airway smooth muscle mass [43,50]. Alternatively, the increased bronchial vasculature represents a protective mechanism through the enhanced clearance of proinflammatory mediators. On the other hand, the enhanced expression of VEGF in the distal airways and alveoli of COPD patients might represent a protective mechanism against the development of emphysema [43,50]. An additional antioxidant function of VEGF in lung parenchyma has been supported by the induction of manganese-superoxide dismutase (MnSOD) expression [51]. These findings suggest that the increased VEGF expression in the distal airspaces may represent a protective mechanism. Collectively, these studies suggest a paradoxical role for VEGF in the bronchi and air spaces in COPD, with a protective function in the alveoli and a detrimental function in the bronchi and bronchioles [50].

### Obstructive sleep apnea

Obstructive sleep apnea (OSA) is associated with recurrent episodes of hypoxia during sleep [25]. The episodes of arterial oxygen desaturation that occur in patients with obstructive sleep apnea have been linked with increased cardiovascular morbidity and mortality [52]. It has been shown that serum and plasma VEGF levels are increased in patients with OSA compared to normal controls [25,53-55]. Circulating VEGF levels significantly correlated with the severity of OSA as expressed by the apnea-hypopnea index [25,53], and are closely correlated to the degree of nocturnal desaturations [55]. It has been suggested that this increase in VEGF levels may represent a response to hypoxia which occurs during sleep [25,53]. Therapeutic interventions, such as oxygen administration during the night and nasal continuous positive airway pressure (CPAP) treatment lead to the reduction of VEGF levels [25,54]. However, no significant differences in circulating VEGF levels were observed in obstructive sleep disordered breathing during childhood [56].

As it has already been mentioned, OSA is associated with considerable cardiovascular morbidity and mortality [52]. However, it has been observed that the risk of the development of cardiovascular disease does not correlate to the severity of OSA [55]. According to this observation, it has been suggested that there might be some unidentified mechanism which protects individual patients with OSA from the development of cardiovascular complications, and VEGF might contribute to this protective mechanism [55]. Nevertheless, it is known that aside from its role in angiogenesis, VEGF may itself take part in the atherogenic process and it has been related to the progression of coronary atherosclerosis in humans [57]. Based on this observation, it has been argued that the augmented VEGF concentration in sleep apnea patients could be a cause of the development of cardiovascular disease by contributing to the atherogenic process itself [25].

### Idiopathic Pulmonary Fibrosis (IPF)

The pathogenesis of IPF is characterized by an initial acute inflammatory reaction which may lead to a chronic fibroproliferative process. The pulmonary architecture is profoundly remodelled, with the extracellular matrix and a variety of cell types involved [58]. Lung biopsies in IPF have the histologic appearance of usual interstitial pneumonia, which is characterized by a heterogeneous and non-uniform fibrosing process with alternating zones of fibrosis, honeycomb change and intervening patches of normal lung.

Plasma VEGF concentrations did not differ between patients with IPF and controls. However, baseline plasma levels of VEGF were significantly related to the extent of parenchymal involvement in HRCT and patients with IPF who developed progressive disease had significantly higher baseline levels of VEGF [59]. In contrast, BAL fluid concentrations of VEGF are significantly depressed in patients with IPF [60-62] and correlate with DL_CO_. The latter correlation possibly reflects the diminished epithelial surface area versus the diminished gene expression or intraluminal secretion of VEGF [60].

The role of VEGF in IPF remains contradictory. A heterogeneity of vascular remodelling in IPF has been reported, with increased vascular density in areas with low grade of fibrosis and decreased vascular density in the most extensively fibrotic lesions [63]. It has been shown that there was an increased expression of VEGF in capillary endothelial cells and alveolar type II epithelial cells in highly vascularized alveolar septa. In contrast, fibroblasts and leukocytes in fibrotic lesions were faintly immunoreactive with VEGF, suggesting a possible role for VEGF in the vascular heterogeneity of IPF [63]. The question according to these findings is whether the increase in vascular density observed in the least fibrotic areas is actively a consequence of the development of the fibrogenic process or represents a compensatory mechanism [64]. The role of VEGF in this process remains to be clarified.

### Sarcoidosis

Sarcoidosis is a multisystem granulomatous disorder of unknown etiology, with frequent pulmonary manifestations, which is often associated with non-granulomatous microangiopathic lesions in various other organs [65]. An increased transcription and protein production of VEGF and an overexpression of its receptor has been found in activated alveolar macrophages, in epithelioid cells, and in multinuclear giant cells of pulmonary sarcoid granulomas [65]. Serum VEGF concentrations were significantly higher in patients who received corticosteroid treatment compared to patients with spontaneous remission. In addition VEGF levels were higher in patients with extrathoracic involvement than in patients in which the disease was limited to the thoracic cage. Based on these findings, the authors suggested that VEGF may represent a marker of disease severity and of extrathoracic involvement in sarcoidosis [66]. In contrast, VEGF levels in BAL fluid from patients with sarcoidosis was significantly lower than normal controls [61]. Low VEGF levels in lung parenchyma may reduce angiogenesis and induce apoptosis of vascular endothelial cells and play a role in the pathogenesis of lung involvement in sarcoidosis.

### Tuberculosis

Circulating VEGF levels are increased in patients with active pulmonary tuberculosis compared to healthy controls and patients with old tuberculosis, and decrease after successful treatment [67,68]. The source of VEGF in pulmonary tuberculosis is believed to be the alveolar macrophages and the CD4 T-lymphocytes [68,69]. Serum VEGF levels were found higher in TB patients without cavitary lesions compared to those with typical chest cavities, suggesting that increased serum VEGF levels may subdue cavity formation [70]. However, this finding was not replicated in subsequent studies [67]. Two studies have reported that VEGF levels may be used for the diagnosis of active tuberculosis, with great sensitivity (93% and 95.8% for cut-off values of 250 pg/mL and 458.5 pg/mL, respectively) but with relatively low specificity [67,68]. VEGF may serve as a marker of disease activity in tuberculosis; however, further studies are needed in this direction.

### Pulmonary hypertension

The pulmonary vasculature exhibits various morphological changes in patients with pulmonary hypertension (PH) [71]. VEGF plays a central role in the life and death of pulmonary vascular endothelial cells [72]. Several reports have suggested a significant role for VEGF in the pathogenesis of PH; however, there are other studies suggesting that VEGF is important in attenuating the development of pulmonary hypertension, possibly by protecting endothelial cells from injury and apoptosis [73].

Plexiform lesions are unique vascular structures that occur in the lungs of patients with primary or secondary PH [74]. VEGF and its receptors flt-1 and flk-1 are expressed in the plexiform lesions and may play a role in the pathogenesis of PH by stimulating dysregulated angiogenesis [71]. In addition, it has been reported that VEGF increases the expression of tissue factor and is likely to play some role in inflammatory responses [72]. Whereas lack of VEGF impaired signaling via the tyrosine kinase receptors causes endothelial cells to die, experimental overexpression of VEGF produces structures that resemble plexiform lesions [72]. Additionally, in animal models with increased pulmonary blood flow and PH the expression of VEGF and its receptors was higher than controls, and that has been suggested to take part in the development of the vascular remodelling seen in PH [75].

In contrast, it has been reported that inhibition of flk-1 in animal models caused pulmonary hypertension characterized by thickening of the medial layer of pulmonary arteries in normoxic conditions. Additionally, in hypoxic conditions, the inhibition of flk-1 lead to more marked pulmonary hypertension developing through an increase in endothelial cell proliferation in the pulmonary artery [76]. These data suggest that VEGF, acting through flk-1, has a protective role and inhibits endothelial cell death [76]. The protective role of VEGF in the development of pulmonary hypertension can also be supported by the fact that VEGF stimulates NO release from vascular endothelium and increases local eNOS expression [77]. Furthermore, it has been shown that gene transfer of VEGF in animal models can reduce bleomycin-induced PH [78].

Finally, it is worthy to mention that platelet VEGF content as well as serum VEGF levels were markedly elevated in patients with primary and secondary PH compared to normal controls, potentially leading to an increase of VEGF at sites of lung injury [79]. Interestingly, platelet VEGF content was further increased by continuous prostacyclin infusion, indicating that prostacyclin increases circulating VEGF levels [79]. The important issue raised from those studies is whether increased platelet VEGF content and potentially increased VEGF released at sites of vascular injury, notably in the pulmonary vasculature, have protective or deleterious effects. The exact role of VEGF in the pathogenesis of human PH and the vascular remodelling inherent in this condition remains unknown [79].

### Pleural effusion

Pleural effusion is a common problem in everyday clinical practice and VEGF has been reported to play an important role in the development of certain types of effusion [24]. Many studies indicate that VEGF is consistently higher in exudative than in transudative pleural effusions [80-83]. Effusions associated with malignancies seem to have higher levels of VEGF than benign effusions [24,81,82,84,85]. Additionally, hemorrhagic malignant effusions presented higher VEGF levels than non-hemorrhagic ones [86,87]. However there are no significant differences in pleural VEGF levels in patients with different histologic types of cancer [82,84], or different clinical stages of lung cancer [82]. VEGF levels in malignant effusions were found to be 10-fold higher than in corresponding serum samples, indicating local release of VEGF within the pleural cavity [88]. It has been suggested that increased VEGF levels in the malignant pleural effusions increases vascular permeability and contributes to fluid accumulation [3,83].

Empyema fluid contains high levels of VEGF, which are significantly higher compared to VEGF levels in uncomplicated parapneumonic effusions [24,89]. It has been suggested that bacterial pathogens induce VEGF release from mesothelial cells and alter mesothelial permeability leading to protein exudation [89]. VEGF levels are higher in tuberculous pleural effusions compared to transudates [90]. In the same study, serum VEGF levels were higher compared to the pleural fluid in patients with tuberculous effusions, implicating that VEGF may promote increased vascular permeability that leads to effusion formation [90]. Finally, Isolated cases of pleural effusions due to pulmonary emboli had very high VEGF levels, probably related to tissue ischemia [81].

Despite the statistically significant differences in pleural fluid VEGF levels between malignant and non malignant effusions, substantial overlap exists, suggesting that VEGF levels are unlikely to be useful diagnostically as a single marker [81]. However it has been proposed that VEGF levels above 1000 pg/ml in pleural fluid are suggestive of either empyema or malignancy [24].

### Lung cancer

VEGF is a potent angiogenic mediator and angiogenesis has important effects on tumor growth and metastasis. Expression of VEGF may therefore be an indicator for the angiogenic potential and biological aggressiveness of a tumor [91]. It has been shown that VEGF [92] and its receptors [93] are expressed in cancer cells, in both non small cell lung cancer (NSCLC) [94] and small cell lung cancer (SCLC) [93]. The molecules of VEGF produced by cancer cells are considered to impact on tumor growth or development via the acceleration of angioneogenesis and lymphangiogenesis and lymph node metastasis [92]. It has been reported that VEGF expression is significantly correlated with neovascularization in resected non small cell lung cancer tissues and can be used as an important prognostic factor [92,95,96]. For instance, VEGF overexpression of in surgically resected adenocarcinomatous lung tissue was indicative of earlier postoperative relapse [97].

Serum VEGF levels are higher in patients with lung cancer than controls [98,99]. In NSCLC, serum VEGF levels were found significantly higher in squamous cell carcinoma than adenocarcinoma [100]. Serum VEGF levels were also significantly associated with the clinical staging of patients with NSCLC [96], while in patients with adenocarcinoma there was a significant correlation of the expression of VEGF_165 _with tumor size [98]. Overall, patients with higher serum VEGF levels had lower survival compared to patients with lower VEGF levels [96,100-103]. The measurement of serum VEGF has also been shown to be a marker of response to chemotherapy, as a decrease of VEGF levels at week 12 after initiation of chemotherapy correlated with response to therapy [102]. In a recent study, pre-treatment VEGF serum levels proved to be an independent prognostic factor in patients with metastatic NSCLC [103]. Lung cancer represents an area where the role of VEGF in prognosis tends to be more established and further therapeutic implications targeting VEGF are already in progress [104].

### Miscellaneous

Measurement of VEGF levels has been a subject of research in several lung diseases. In cystic fibrosis elevated serum VEGF levels were found and were further increased during pulmonary exacerbations [105]. VEGF levels in BAL fluid of patients with acute eosinophilic pneumonia are higher than normal controls and rapidly decrease to the control level with clinical improvement; these findings suggest an important role for VEGF in the pathogenesis of pulmonary edema in eosinophilic pneumonia [106]. VEGF levels are also increased in BAL fluid, serum and tissue of patients with hypersensitivity pneumonitis, suggesting that abnormal expression of VEGF may contribute to impair the lung repair in this disease [107].

### Therapeutic implications and perspectives

The fact that VEGF levels correlated with cancer staging and prognosis, has supported the idea of using anti-VEGF strategies, such as anti VEGF antibodies (e.g. bevacizumab) or inhibitors of the VEGF receptors in combination with chemotherapy or alone to improve survival of patients with metastatic NSCLC [103,108]. Generally tumors cannot grow beyond 2 mm in diameter without developing vascular supply. Neovascularization permits further growth of the primary tumor, but it also provides a pathway for migrating tumor cells to gain access to the systemic circulation and to establish distant metastases [109]. As VEGF and its receptors play an important role in tumor growth and metastasis, the use of anti-VEGF agents and VEGF-R inhibitors for the treatment of lung cancer is currently in development, and bevacizumab is the first anti-VEGF factor that has already been used in patients with lung cancer [110].

RhuMab VEGF, is a recombinant humanized monoclonal antibody to VEGF that has been shown to inhibit the growth of a variety of human cancer cell lines [111]. This agent may act synergistically with chemotherapy and is currently being tested in lung cancer. The VEGF system can also be targeted through inhibition of VEGFR, by the use of monoclonal antibodies or specific tyrosine kinase inhibitors [111]. Currently studied inhibitors of VEGFR include SU5416 (a VEGFR-2 inhibitor) and SU6668 (a VEGFR-1 inhibitor). Although SU5416 suppresses tumor growth in animal models [112], neither of these agents will be developed further in view of their adverse toxicity profile [111]. Other inhibitors such as ZD6474, and CP-547,632 are still under research [111].

Blockade of VEGF activity in malignant pleural effusions has been proposed as an intervention to decrease permeability and reduce pleural fluid [3,113]. On the other hand, in animal models where pleurodesis was induced with TGF-β_2_, treatment with anti-VEGF antibody before TGF-β_2 _injection resulted in decrease of the amount of angiogenesis and inhibition of pleurodesis [114]. As agents that act as anti-VEGF agents are now being used in the treatment of several different tumors, one should probably not attempt to perform pleurodesis when the patient has already been receiving an agent that inhibits angiogenesis [114].

The use of VEGFR-2 inhibitors has been proposed as additional therapy for patients with progressive pulmonary fibrosis [59]. However, other investigators have reported that antagonizing VEGF would not be a successful potential treatment for patients with pulmonary fibrosis as they suggest that this would hasten epithelial cell apoptosis and promote alveolar septal cell loss resulting to honeycombing and functional deterioration [115].

## Conclusion

Conclusively, the answer to the question "friend or foe" for VEGF in the lung is not an obvious one. VEGF may have a protective role in specific areas of the lung and a deleterious role in other areas, being part of a procedure which leads to damage. The lack of VEGF in some disease entities may provide an indication for its substitution, whereas its overexpression in other pathological conditions has led to efforts for blockage of its actions. The only possible answer that could be given is that VEGF in the lung could be a good friend as long as it is present in the right amount, in the right place and in the right time. Further research is still needed for the complete understanding of the exact role of VEGF in health and disease, in order to take advantage of its benefits and avoid its adverse effects.

## Competing interests

The author(s) declare that they have no competing interests.

## Authors' contributions

AP and KK were involved in the study conception. AP, KK and PK performed the data acquisition and interpretation. AP prepared the manuscript. KK and KG were involved in revising the manuscript for important intellectual content. All authors read and approved the final manuscript.
